# Correlates of mental health stigma in the Buyende district of Eastern Uganda

**DOI:** 10.1017/gmh.2026.10269

**Published:** 2026-07-02

**Authors:** Ethan Morgan Chang, Jaimie Almaraz, Ryan Alezz, Kayera Sumaya Nakaziba, Job Basiimwa, Tubenawe Bruno, Shakirah N. Lieffers, Travor Nkolo, Junior Frimpong, Robert Rosenheck, Yang Jae Lee

**Affiliations:** 1 Colby College, USA; 2 Tufts University School of Medicine, USA; 3 Johns Hopkins University, USA; 4 Empower Through Health, Uganda; 5 Cavendish University Uganda, Uganda; 6 University of Michigan, USA; 7 The University of Tennessee Health Science Center College of Medicine, USA; 8 Yale University, USA; 9 Department of Psychiatry and Behavioral Sciences, University of Washington School of Medicine, USA; 10 Department of Global Health, University of Washington, USA

**Keywords:** stigmatization, mental health, mental illness, gender, religion

## Abstract

Understanding correlates and potential causes of mental illness stigma in community-based settings in low- and middle-income countries (LMICs) is important for developing effective interventions to reduce stigma and demand-side barriers for treatment. This study analyzed data from structured questionnaires administered to 178 respondents in the Buyende district in Eastern Uganda to investigate sociodemographic and clinical factors correlated with mental illness stigma. Factor analysis of 33 items revealed a single dominant factor reflecting mental health stigma. Bivariate and multivariate analyses of sociodemographic and clinical correlates were used to identify factors associated with stigma in the entire sample and separately within the subgroups with evidence of mental illness. In the entire sample, female gender was the only independent correlate of stigma. Analysis of the mental illness subgroup also showed that women had high levels of mental illness stigma. These findings suggest that female gender appears to be a more important correlate of mental health stigma than clinical factors. Nevertheless, effective destigmatizing interventions are needed for the entire population, with additional approaches specifically tailored to women.

## Impact statement

Mental health stigma has remained a major barrier to mental health services in low- and middle-income countries (LMICs). Rural settings within LMICs are especially vulnerable due to a lack of formal mental health services. This study identified female gender as a correlate of mental illness stigma, a group that may require targeted stigma interventions to address their distinct risk factors. This study investigated sociodemographic and clinical factors of mental health stigma within the Buyende District of rural Eastern Uganda to identify distinct dimensions and correlates of stigma. Notably, we identified the female gender as the strongest correlate of mental health stigma, even among the subgroup with evidence of mental illness. These findings support previous research that identifies gender as a major stigma correlate and highlights the importance of cultural context in mental health interventions. These results help develop an understanding of mental health stigma within LMICs, especially the role of gender as a barrier to mental health interventions within rural Eastern Uganda. Furthermore, our results provide insight that can be used by policymakers and mental health practitioners to develop interventions to improve mental health outcomes by reducing stigma and demand-side barriers for treatment in LMICs.

## Introduction

Globally, it is estimated that one in eight people lives with a mental disorder, with low and middle-income countries (LMICs) bearing more than 80% of the global burden of mental illness (Rathod et al., 2017). Despite the predominant burden of mental illness in LMIC countries, relevant health systems have yet to adequately address the needs of people with mental disorders (World Health Organization, 2016). In many LMICs, including Uganda, the focus of this report, less than 1% of health budgets are allocated toward mental health services, and the supply of trained professionals is severely limited (Rathod et al., 2017).

While financial and personnel resource limits pose a major impediment to treatment availability, stigma toward people with mental disorders imposes a major additional barrier to the use of services (Knaak et al., 2017). Mental health stigma is defined as negative attitudes, stereotypes and beliefs that people express toward those with mental health conditions. Stigma has the possibility of creating fear, exclusion and a reluctance to seek care (Knaak et al., 2017; Habeb et al., 2025). Both cultural and personal factors play a role in shaping mental health stigma, influencing self-perception (as well as public perception) and behavior toward people with mental illnesses (Javed et al., 2021). Vulnerable groups, such as women, ethnic minorities and the economically disadvantaged, are reported to be more affected by stigma than others (Mascayano et al., 2015; Molodynski et al., 2017). A global 2023 study, for example, found that perinatal women seem to avoid seeking mental health care to protect their right to infant care and community acceptance (Pokharel et al., 2023). Stigmatized groups may anticipate public scrutiny and discrimination, leading to further treatment avoidance (Mascayano et al., 2015).

However, large variations even among vulnerable groups, such as women, exist across cultures. In South India, women reported more perceived mental illness stigma than their male counterparts (Kulesza et al., 2014). Women in Pakistan also experienced greater levels of internalized stigma compared to men (Khan et al., 2015). In a vignette-based study in rural Uganda, women with depression were perceived as less stigmatized than men in a mixed gender sample of respondents (Lee et al., 2024b). A 2019 study in rural Uganda found that women with mental illness were thought to be a disgrace to their families, and interviewees reported unwillingness to have women with mental illness marry into their families (Rasmussen et al., 2019). These differences underscore the importance of more closely examining a wide range of correlates of stigma in rural communities, including indicators of mental health status, as well as the investigation of multiple dimensions of stigma that may influence perceptions of mental health.

Understanding correlates and potential causes of stigma in community-based settings may be an important step in developing interventions to reduce stigma in LMICs (Mascayano et al., 2015). Nationally, in Uganda, mental healthcare coverage is not widely accessible at a community level (Molodynski et al., 2017). Stigma related to mental illness may represent an additional barrier to seeking biomedical care in rural Uganda (Lee et al., 2026). As a result, at the local level, people may be more likely to choose traditional healers before seeking evidence-based treatment from biomedical providers (Bwanika et al., 2022; Lee et al., 2025a, 2024c). Though various studies have examined mental illness stigma in Uganda, the specific association of stigma with sociodemographic characteristics in rural communities, such as gender, age and religious affiliation, along with mental health diagnoses and psychiatric symptoms, remains understudied.

In this study, we seek to evaluate the association of sociodemographic characteristics along with mental health diagnostic and symptom indicators within a unidimensional dimension of social distancing stigma in rural Eastern Uganda. We aim to better understand underlying factors associated with mental illness stigma and investigate variation in stigma across population groups, particularly among individuals with evidence of mental illness. Identification of these patterns will aid in the creation of intervention strategies to reduce stigma and improve the utilization of mental health services in rural Uganda while contributing to the broader understanding of stigma in LMICs.

## Methods

### Study site

**Figure 1.**
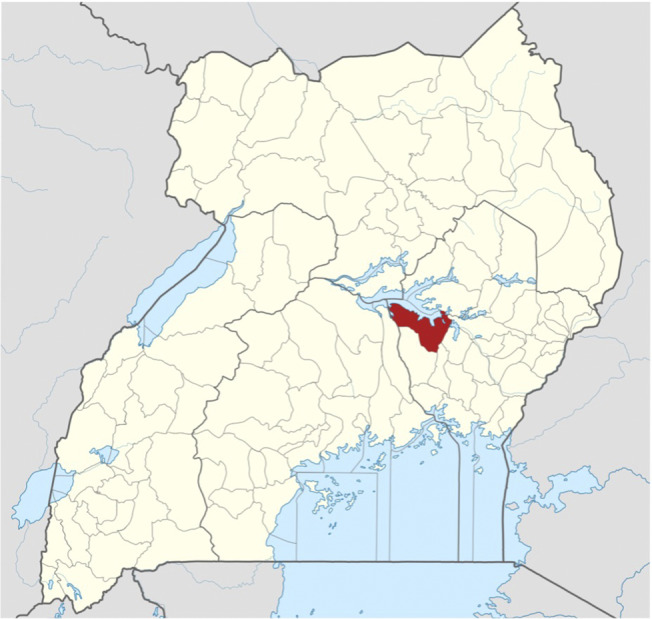
Buyende district of Uganda (OpenStreetMap et al., 2017).

The study was conducted in the Buyende District in the Busoga region of Eastern Uganda (Figure 1). The Buyende district has a population of 403,486 and is primarily rural, characterized by 46.9% of households living within a subsistence economy and a 15.7% unemployment rate (Uganda Bureau of Statistics, 2024). Previous studies have shown complex patterns of care-seeking for mental illnesses and physical illnesses in the region (Lee et al., 2019a, 2019b, 2025b). Findings from these studies indicate that individuals often combine traditional and biomedical treatment, seek care from informal providers and may receive treatment that does not align with Ugandan Ministry of Health guidelines (Lee et al., 2019a, 2019b, 2025b).

### Study design and sampling

General community participants were recruited via convenience sampling from villages within the study area. The study was introduced to local leadership and Village Health Team (VHT) members. VHTs in Uganda are community volunteer health workers who are trained to promote and provide basic health education and serve as a link between households and the formal health system (Ministry of Health, 2010). Plain language descriptions of common mental illnesses were provided to potential respondents to clarify what was meant by mental illness. To assure representation of individuals with mental illness, additional respondents were recruited from Empower Through Health’s (ETH) existing patient cohort at Mpunde Health Center and via referrals from VHTs. VHTs referred respondents with mental illness based on existing diagnoses by Ugandan healthcare professionals. Participants were only invited to participate if they had depression, anxiety, schizophrenia, substance use and psychosis diagnosis(es).

### Participants: Inclusion and exclusion

Eligible participants reported age ≥18 years and were deemed capable of providing informed consent and were fluent in English, Lusoga or Luganda (the two most common local languages). Individuals were included in the mental illness group if they met the criteria on one of five screening tools described below. All people diagnosed with a mental illness through the Empower Through Health clinic and VHTs underwent the psychiatric symptom screening for continuity. We attempted to reach a threshold of *n* = 50 for the Mental Illness cohort for statistical validity. Exclusion criteria included inability or unwillingness to provide informed consent or reported age < 18 years.

### Informed consent and participant protections

All participants underwent an informed-consent process prior to any study activities. Verbal consent was obtained to assess eligibility. Written consent was then obtained from eligible participants. For participants who were illiterate, a witnessed thumbprint documented consent. Participants identified with a probable mental illness were referred for clinical care after interview completion. Participants received compensation for time and transport. Concurrent treatment was not an exclusion criterion.

### Field team and procedures

Interviews were conducted by experienced research assistants (RAs) who possessed working proficiency in English and Lusoga or Luganda and had prior experience working on research projects in the Buyende district. RAs obtained written informed consent, explained confidentiality protections, administered all survey modules and compiled data for central analysis. They also administered screening measures described below and Mini-International Neuropsychiatric Interview (M.I.N.I) modules in keeping with standard practice for these measures, as described in the relevant literature (Sheehan et al., 1998; Korte et al., 2023). The M.I.N.I. is a brief, structured diagnostic interview for the Diagnostic and Statistical Manual of Mental Disorders (DSM-IV) and the International Classification of Diseases (ICD-10) psychiatric disorders with strong international validity and established use in Ugandan research settings (Sheehan et al., 1998; Korte et al., 2023). The M.I.N.I. psychosis module (Module K) was administered by a trained clinician.

### Measures

#### Stigma items

Thirty-three stigma items were available for all participants from three sources:

- Ten items from the Broad Acceptance Scale, a questionnaire designed to reflect factors that contribute to structural stigma (Lee et al., 2022, 2024a).
- Thirteen items from the distal stigma questionnaire, which included four items derived from the Community Attitudes Toward the Mentally Ill (CAMI) scale and nine items from a previous study conducted by the research team (Taylor and Dear, 1981; Lee et al., 2022).
- Ten items from the attributed stigma questionnaire, developed from the Personal Acceptance Scale (designed to reflect factors that contribute to social/public stigma) (Lee et al., 2022).

These items to assess stigma were drawn from methods employed by previous research teams investigating similar constructs and varying dimensions of stigma in Uganda using dichotomous items (Lee et al., 2022, 2024a). Items were selected to maintain consistency with established methodologies.

#### Psychiatric symptom screening

Five screening instruments, four with Uganda-specific validation, addressing diverse dimensions of mental illness, following published algorithms and cut points are as follows:

- **Depression**: Patient Health Questionnaire (PHQ-9). A positive algorithm screen was defined as ≥4 shaded responses including items 1 or 2 (Kroenke et al., 2001). Ugandan validation supports use of the PHQ-9 (Nakku et al., 2016; Miller et al., 2021).
- **Generalized anxiety**: Generalized Anxiety Disorder 7-item scale (GAD-7), with a positive screen at ≥10 (Spitzer et al., 2006). Recent psychometric work supports the GAD-7 in Ugandan language contexts (Ziegel et al., 2025).
- **Bipolar spectrum**: Mood Disorder Questionnaire (MDQ) using standard scoring (≥7 “Yes” on Q1, “Yes” on Q2 and “Moderate/Serious” on Q3) (Hirschfeld et al., 2000). (Uganda-specific validation remains unavailable.)
- **Psychosis**: Psychosis Screening Questionnaire (PSQ); a positive screen required endorsement of a root item plus ≥1 corroborating probes (Bebbington and Nayani, 1995). Construct validity in Ugandan adults has been reported (Kwagala et al., 2023).
- **Alcohol use**: Alcohol Use Disorders Identification Test (AUDIT) with a cutoff of ≥8 (Saunders et al., 1993). Validation against multiple reference standards has been demonstrated in Ugandan cohorts (Kuteesa et al., 2019).

Participants who screened positive on any of these five instruments completed the relevant M.I.N.I. module(s) for diagnostic categorization (Module A after PHQ-9+, Module C after MDQ+, Module I after AUDIT+, Module K after PSQ+ and Module N after GAD-7+). If participants screened positive on more than one instrument, they completed all M.I.N.I. modules relevant to their positive screens. If a participant screened positive on the PHQ-9, GAD-7, MDQ, PSQ and/or AUDIT but tested negative on all M.I.N.I modules, the participant was placed into the nonmental illness cohort.

#### Data collection and management

Questionnaires were administered in English, Lusoga, or Luganda according to participant preference. RAs entered responses directly into KoBoToolbox on password- protected Android tablets. KoBoToolbox is an open-source data-collection platform widely used in low-resource and humanitarian settings (KoBoToolbox, 2026). Entries were encrypted, and routine backups were created and stored securely. Paper consent forms were kept in locked storage at the field office. Personally identifiable information remained in Uganda.

#### Demographic data

All Participants were asked to report age, sex, marital status, highest level of completed education and religion. All participants were asked to report any diagnosis of depression, anxiety, schizophrenia, substance use and psychosis. Participants diagnosed with unknown or unlisted mental illnesses were asked to specify.

### Analysis

#### Factor analysis

First, to understand social stigma, an exploratory factor analysis was conducted on the 33 stigma items among respondents without evidence of mental illness (*n* = 126). Four items with zero variance were excluded, leaving 29 items for analysis. One respondent had missing data for four of the 29 items (Most People Not Next Door, Most People Fear Neighbors, Most People No Sympathy, Rev. Most People Trust Children). We substituted the missing responses using mean substitution. The number of factors to retain was evaluated using parallel analysis and Velicer’s minimum average partial (MAP) test, which initially suggested a multifactorial structure (parallel analysis: 5 factors; MAP: 4 factors). Examination of the three-factor varimax solution, however, revealed that factor composition was strongly associated with item wording direction. Among items with loadings greater than or equal to 0.4, Factor 2 consisted entirely of reverse-worded items (7/7; 100%), Factor 1 was predominantly nonreverse-worded (9/11; 82%) and Factor 3 was evenly split (4/8; 50%). This pattern suggested that factors were defined substantially by wording method rather than the substantive content.

To test this interpretation, we conducted separate factor analyses within each wording type. Velicer’s MAP suggested no additional factors beyond a general factor for both reverse-worded items (*n* = 12; Cronbach’s alpha = 0.78) and nonreverse-worded items (*n* = 17; Cronbach’s alpha = 0.78), consistent with unidimensionality within wording type. Given (1) the alignment between factor membership and item wording direction, (2) high overall internal consistency (Cronbach’s alpha = 0.86) and (3) theoretical coherence of all items as measures of social distancing in response to stigma, we interpret the scale as essentially unidimensional. Factor loadings and wording classifications for all items appear in Supplementary Appendix Table A1.

To investigate if similar factor loadings were present in the mental illness cohort (*n* = 52), an exploratory factor analysis with the 29 available items was completed to investigate internalized stigma using the same methodology as above. A scree plot and parallel analysis suggested a three-factor solution. Factor loadings can be found in Supplementary Appendix Table A2. Among factor loadings greater than or equal to 0.4, similar patterns of wording method bias were observed in the three-factor model. Therefore, we conducted a one-factor exploratory factor analysis to compare factor loadings with the non-MI subgroup. Unidimensionality was observed between the two groups with minimal deviance in factor loadings between the MI and non-MI factors. Therefore, we believed that a pooled one-factor exploratory factor analysis was suitable for final analysis.

Finally, a unidimensional exploratory factor analysis with the 29 available items was completed on the entire sample (*n* = 178). Items with factor loadings ≥0.45 were included in the created social distancing factor. Factor loadings can be found in Table 1. This cutoff was chosen to reduce the role of sample size and overdetermination in accordance with previous literature (MacCallum et al., 1999). Internal consistency was assessed for the factor using Cronbach’s alpha.

**Table 1.** Unidimensional factor analysis (without rotation) of items reflecting stigma toward people with mental illness, including factors with weights greater than 0.45 and average item scores for each group on the overall factor^#^ Table 1. long description.

Variables	Non-MI	MI	Combined
Indicates an willingness to work with someone with a mental illness*	0.52	0.51	0.51
Indicates willingness to have casual conversations with neighbors who suffer from mental illness*	0.46	0.49	0.46
Finds it frightening to think of mentally ill people being neighbors	0.53	0.53	0.53
Indicates a belief that most women who were patients in a mental hospital can be trusted to watch children*	0.59	0.59	0.59
Believes people with mental illness are a public nuisance	0.51	0.49	0.49
Believes it is foolish for a woman to marry a man who has recovered from mental illness	0.46	0.46	0.46
Believes people with mental illness are a burden on society	0.53	0.50	0.50
Indicates a belief that more emphasis should not be placed on protecting the public from mentally ill people*	0.45	0.45	0.45
Indicates a belief that people with mental health problems should have equal job rights*	0.58	0.60	0.60
Believes most people are afraid of people with mental illness	0.52	0.50	0.50
Believes most people would object to mentally ill people living in their neighborhood	0.57	0.54	0.54
Indicates a belief that most people would invite mentally ill individuals into their home*	0.53	0.54	0.54
Indicates a belief that most people would have casual conversations with mentally ill neighbors*	0.50	0.52	0.52
Believes most people find it frightening to think of people with mental problems being their neighbors	0.55	0.55	0.55
Believes most people believe that most women who were once patients in a mental hospital can be trusted to watch their child*	0.66	0.63	0.63
**Average factor score** **	**0.47**	**0.39**	**0.44**

Factor Labels: **Combined** = Combined data (*n* = 128); **MI** = Those with indication of mental illness (*n* = 52); **Non-MI** = Those without indication of mental illness (*n* = 126).

# Fifteen items that did not load on the combined factor with a weight > 0.45 included: Afraid Mental Illness, Object Neighborhood, Avoid Conversations, Invite to Home*, Not Next Door, No Sympathy, No Responsibility of Care, Exclude Public Office, Neighborhood Fearless*, Exclude Neighborhood*, General Avoid Conversations, General Work Willingness*, Most People Not Next Door, Most People No Sympathy.

* Reverse-coded items.

** Average item score of the items loading over 0.45 for the overall factor, among all respondents.

#### Linear regression and bivariate Pearson correlation

Next, a bivariate analysis of the entire sample was used to compare respondents who met screening criteria for a mental disorder, confirmed by the M.I.N.I., and those who did not on sociodemographic characteristics and the dimension of social distancing stigma identified through the factor analysis described above. Multivariable linear regression was then used to identify significant differences between these groups on the stigma measures, adjusting for any significantly different sociodemographic characteristics.

A bivariate Pearson correlation was used to identify significant correlates of the stigma measures with sociodemographic characteristics, continuous symptom measures available from the five screening measures and both self-reported diagnoses and diagnostic categorization based on the M.I.N.I. Finally, a stepwise multivariate linear regression was used to identify the independent relationship between stigma items and characteristics found to be significant on bivariate analysis.

## Results

Data from 178 respondents were available for factor analysis. Four participants screened positive on the initial screeners but tested negative on the M.I.N.I. These participants were placed into the community subgroup. Demographic data of all respondents revealed 52.8% identifying as female and a mean age of 42.5 (SD: 15.3). Differences in demographics between the mental illness and nonmental illness were not statistically significant, except for gender (*p* < 0.001). Further demographic data can be found in Table 2. Orthogonal factor analysis (Table 1) revealed a unidimensional social distancing factor with 15 items. Cronbach’s alpha showed adequate internal consistency (0.85), and the factor accounted for 19.1% of variance. Bivariate comparison of respondents with and without mental illness (*N* = 52 and *N* = 126, respectively) showed significant differences in gender, with fewer females in the group with mental illness (26.9% vs. 63.9%, *p* < 0.001). Higher stigma scores were observed in the nonmentally ill group (*p* = 0.03) (Table 2). In multiple regression analysis of mental illness indication and stigma, controlling for gender, the factor was no longer significant (*p* = 0.15), suggesting gender as a confounding variable. An interaction analysis of gender and mental illness in association with personal distancing stigma was significant (*t* = 3.32, *df* = 174, *p* = 0.001, *B* = 0.261), indicating that men with mental illness had significantly lower personal stigma values than all other respondents, and reciprocally that women with mental illness had higher stigma scores than the other groups.

**Table 2.** Comparisons of respondents with mental illness and without mental illness (Chi-square and t-tests) Table 2. long description.

Categorical variables	Total*N* = 178	Mental illness *n* = 52	Non-mentalIllness *n* = 126		
	*n* (%)	*n* (%)	*n* (%)	*df*	*χ^2^*
Gender				1	18.3***
Female	94 (52.8)	14 (26.9)	80 (63.5)		
Male	84 (47.2)	38 (73.1)	46 (36.5)		
Marital status				3	1.08
Separated	18 (10.1)	7 (13.5)	11 (8.7)		
Single and never married	7 (3.9)	2 (3.8)	5 (4.0)		
Married or cohabiting	141 (79.2)	39 (75.0)	102 (81.0)		
Widowed	12 (6.7)	4 (7.7)	8 (6.3)		
Education				4	2.71
Never attended	24 (13.5)	6 (11.5)	18 (14.3)		
Incomplete primary	104 (58.4)	35 (67.3)	69 (54.8)		
Complete primary	32 (18.0)	7 (13.5)	25 (19.8)		
O-Level (completed HS)	17 (9.5)	4 (7.7)	13 (10.3)		
Vocational training	1 (0.6)	0 (0.0)	1 (0.8)		
Religion				1	0.02
Christian	150 (84.3)	43 (82.7)	107 (84.9)		
Muslim	28 (15.7)	9 (17.3)	19 (15.1)		
Continuous variables	*M (SD)*	*M (SD)*	*M (SD)*	*df*	*t*
Age (years)	42.5 (15.3)	45.7 (16.2)	41.2 (14.8)	87	1.75
Stigma factor Social distance	0.44 (0.23)	0.39 (0.21)	0.47 (0.23)	93	1.68

*Note*: *p < 0.05; **p < 0.01; ***p < 0.001.

Further examination of the group identified as having mental illness (*N* = 52) (Table 3) showed a significant positive correlation between female gender and the social distancing factor (*t* = 4.37, *df* = 50, *p* < 0.001, *B* = 0.556). Self-reported diagnosis of schizophrenia (*t* = 2.293, *df* = 50, *p* = 0.03, *B* = 0.308) and M.I.N.I. diagnosis of anxiety (*t* = 2.27, *df* = 50, *p* = 0.03, *B* = 0.305) were both significantly correlated with the factor. A significant negative correlation was also observed between the social distancing factor and PSQ score (*t* = −2.97, *df* = 50, *p* = 0.005, *B* = −0.387).

**Table 3.** Correlation of categorical and continuous variables for respondents with mental illness Table 3. long description.

Categorical variables	Mental illness *n* = 52	Social distance factor
	*n* (%)	*r*
Gender		0.56***
Female	14 (26.9)	
Male	38 (73.1)	
Marital status		
Separated	7 (13.2)	
Single and never married	2 (3.8)	
Married or cohabiting	39 (73.6)	−0.03
Widowed education	4 (7.6)	
Never attended	6 (11.5)	
Incomplete primary	35 (67.3)	0.02
Complete primary	7 (13.5)	
O-Level	4 (7.70)	
Vocational training	0 (0.0)	
Religion		−0.021
Christian	43 (82.7)	
Muslim	9 (17.3)	
Diagnoses		
Depression	23 (44.2)	0.03
Anxiety	10 (19.2)	−0.127
Schizophrenia	3 (5.8)	0.30*
Substance use	15 (28.8)	0.03
Psychosis	2 (3.8)	−0.12
Other	1 (1.9)	−0.08
Unknown	1 (1.9)	0.05
M.I.N.I diagnoses^†^		
Alcohol use	29 (53.8)	−0.08
Depression	25 (48.1)	−0.02
Anxiety	19 (36.5)	0.305*
Psychosis	10 (19)	−0.03
Mania	9 (17.5)	−0.05
Schizophrenia	2 (4.0)	0.21
Schizoaffective	1 (2.0)	0.10
Mood	9 (17.5)	−0.05

Continuous variables	*M (SD)*	Social Distance Factor
Age (years)	45.7 (16.2)	−0.22
Psychometric screening tools^‡^		
AUDIT Score	14.5 (12.5)	0.02
PHQ–9 Score	13.0 (6.44)	0.07
MDQ Score	0.34 (0.48)	−0.19
GAD–7 Score	10.5 (5.4)	−0.07
PSQ Score	0.58 (0.50)	−0.39**

*Note*: *p < 0.05; **p < 0.01; ***p < 0.001.

† M.I.N.I.: Mini International Neuropsychiatric Interview.

‡ Psychometric screening tools: AUDIT: Alcohol Use Disorders Identification Test; PHQ-9: Patient Health Questionnaire-9; GAD-7: Generalized Anxiety Disorder-7; MDQ: Mood Disorder Questionnaire; PSQ: Psychosis Screening Questionnaire.

In the stepwise multiple regression analysis, sex, M.I.N.I. diagnosis of anxiety and PSQ score were retained in the final model (F (3,48) = 12.58, *p* < 0.001, *R* ^2^ = 0.44). The self-reported schizophrenia diagnosis was excluded during stepwise selection.

## Discussion

In this small rural Eastern Uganda sample, stigma-related items clustered into a social distancing factor. This suggests that our factor is a measure of social distancing stigma, related to intimate relationships such as caring for one’s child, work environments and proximity of living. Across analyses, gender emerged as the most consistent correlate of stigmatizing attitudes within this exploratory study. In the overall sample, an apparent difference in stigma between respondents with and without evidence of mental illness was attenuated after adjustment for gender. An interaction analysis suggested the lowest social distancing stigma was concentrated among men with evidence of mental illness, while women with evidence of mental illness reported comparatively higher social distancing stigma. Within the subgroup with evidence of mental illness, female gender remained positively associated with social distancing in multivariable models, underscoring gender as the primary respondent characteristic associated with the stigma factor.

In secondary analyses incorporating additional symptom and diagnostic measures available in the subgroup with evidence of mental illness, clinical indicators showed weak and less consistent associations with social distancing stigma than did gender. Specifically, psychotic symptomatology showed a modest inverse association with the factor. If applying a Bonferroni correction across the stigma dimension (*p* < 0.02), the associations with gender and affiliation remained statistically significant, whereas the association with psychotic symptomatology did not.

### Comparison with previous literature

Our findings that women are a correlate of mental illness stigma are consistent with the limited previous research from LMICs (Venkatesh et al., 2015). Although a previous report from rural Uganda reported less stigma toward women than men when presented with a vignette of a depressed woman as compared to a vignette of a depressed man, the current study examines stigma toward mental illness at a general level (Lee et al., 2024b). As such, our findings should be interpreted as a reflection of overall perceptions, rather than diagnosis-specific stigma. In our effort to understand the findings of this study, we believe that the observed gender difference in expressed stigmatized attitudes may stem from specific dependent roles of women in rural Ugandan society, as in traditional cultures in other LMICs (Sharma et al., 2016). The Economic Policy Research Center reports that Ugandan women are reported to spend disproportionately more time per week in unpaid roles such as cooking and childcare, making them dependent on and subordinate to men and thus more vulnerable to adversity if men are disabled by mental illness (Mwesigwa, 2022). Examples of social consequences in LMICs are further exemplified in a South-Central Ethiopian study, which found women with male partners who had serious mental illness were expected not to divorce their husbands, thus making them exceptionally vulnerable, an expectation that was not applied to men (Hailemariam et al., 2019). A plausible interpretation of our findings is thus that mental illness creates large strains for female social roles, leaving women more vulnerable to adverse consequences if their male partners have disabling mental illnesses. This interpretation is consistent with findings that gender and societal role expectations drive higher levels of stigmatization of mental illness by women than by men in South India (Venkatesh et al., 2015).

### Implications for clinical practice and policy

Stigma is recognized as an important impediment to treatment seeking, especially in LMICs and rural areas. The results of this study suggest that efforts to reduce stigma may be somewhat more urgent for women than men; however, further research is needed to understand our findings. Nevertheless, the data reported here should not be taken as an indication that efforts to reduce stigma should focus especially on women. Rather, such efforts should be focused on all segments of the population in rural areas and LMICS, with the development of special messaging for women.

### Limitations

The primary methodological limitations of this study concern the relatively small sample size and the recruitment of a convenience sample of respondents from both the general population and from a clinic providing mental health services. It was notable, however, that despite the limited sample size, several significant relationships were observed, even after adjustment for multiple comparisons, as well as adjustment for significant sociodemographic differences between respondents with and without evidence of mental illness. However, these findings need to be confirmed in a larger, more representative sample.

Another limitation is that the measures of stigmatizing attitudes refer to mental illness, in general, rather than to specific mental disorders. Previous research has shown differences in stigmatizing attitudes toward different mental disorders (Lee et al., 2024b). However, addressing attitudes toward specific disorders would have posed considerable additional respondent burden and thus was beyond the scope of this study.

Among respondents with evidence of mental illness, the single factor likely reflects internalized stigma. This is contrasted by the respondents without mental illness, in which the same items reflect public stigma. Our pooled analysis combines related but distinct stigma dimensions, which we cannot separate, given the current design. Furthermore, the use of standard EFA methods for dichotomous items is a limitation in the interpretation of a unidimensional structure. Thus, unidimensionality should be interpreted cautiously. Future research should distinguish between internalized and public stigma by incorporating measures designed to assess public stigma in all sociodemographic factors.

Finally, the small sample size of the respondents with evidence of mental illness implies that multivariate findings are exploratory and should be interpreted cautiously. Thus, findings in the mental illness subgroup should be confirmed through further research utilizing a larger, more representative sample.

## Conclusion

This study of stigma toward people with mental illness in rural Uganda showed that the strongest associations of stigma with any respondent characteristic were observed with female gender, with limited evidence of associations with specific clinical factors.

Nevertheless, our judgment is that antistigma initiatives should be directed broadly to the entire population, albeit with consideration of messaging specifically appealing to women.

## Supporting information

10.1017/gmh.2026.10269.sm001 Chang et al. supplementary material 1 Chang et al. supplementary material

10.1017/gmh.2026.10269.sm002 Chang et al. supplementary material 2 Chang et al. supplementary material

## Data Availability

Raw data are not available to the public.

## Supplementary material

The supplementary material for this article can be found at http://doi.org/10.1017/gmh.2026.10269.

## Long descriptions

### Table 1. Long description

The table consists of four columns: Variables, Non-M I, M I, and Combined. It lists 15 items with factor weights greater than 0.45.

* Willingness to work with someone with a mental illness: Non-M I 0.52, M I 0.51, Combined 0.51.

* Willingness to have casual conversations with neighbors: Non-M I 0.46, M I 0.49, Combined 0.46.

* Finds it frightening to think of mentally ill neighbors: Non-M I 0.53, M I 0.53, Combined 0.53.

* Belief that former mental hospital patients can be trusted to watch children: Non-M I 0.59, M I 0.59, Combined 0.59.

* Believes people with mental illness are a public nuisance: Non-M I 0.51, M I 0.49, Combined 0.49.

* Believes it is foolish for a woman to marry a man recovered from mental illness: Non-M I 0.46, M I 0.46, Combined 0.46.

* Believes people with mental illness are a burden on society: Non-M I 0.53, M I 0.50, Combined 0.50.

* Belief that more emphasis should not be placed on protecting the public: Non-M I 0.45, M I 0.45, Combined 0.45.

* Belief that people with mental health problems should have equal job rights: Non-M I 0.58, M I 0.60, Combined 0.60.

* Believes most people are afraid of people with mental illness: Non-M I 0.52, M I 0.50, Combined 0.50.

* Believes most people would object to mentally ill people living in their neighborhood: Non-M I 0.57, M I 0.54, Combined 0.54.

* Belief that most people would invite mentally ill individuals into their home: Non-M I 0.53, M I 0.54, Combined 0.54.

* Belief that most people would have casual conversations with mentally ill neighbors: Non-M I 0.50, M I 0.52, Combined 0.52.

* Believes most people find it frightening to think of people with mental problems being neighbors: Non-M I 0.55, M I 0.55, Combined 0.55.

* Believes most people believe former mental hospital patients can be trusted to watch children: Non-M I 0.66, M I 0.63, Combined 0.63.

* Average factor score: Non-M I 0.47, M I 0.39, Combined 0.44.

Navigate back to Table 1..

### Table 2. Long description

The table is divided into two main sections: Categorical variables and Continuous variables.

Categorical variables section includes:

- Gender: Total N equals 178. Female 94 (52.8 percent), Male 84 (47.2 percent). Mental illness group (n equals 52) is 26.9 percent female and 73.1 percent male. Illness group (n equals 126) is 63.5 percent female and 36.5 percent male. Chi-square equals 18.3 with p less than 0.001.

- Marital status: Majority are Married or cohabiting (79.2 percent total). Chi-square equals 1.08, d f equals 3.

- Education: Majority have Incomplete primary education (58.4 percent total). Chi-square equals 2.71, d f equals 4.

- Religion: Christian (84.3 percent total) and Muslim (15.7 percent total). Chi-square equals 0.02, d f equals 1.

Continuous variables section (Mean and S D):

- Age (years): Total Mean 42.5 (S D 15.3). Mental illness group Mean 45.7 (S D 16.2). Illness group Mean 41.2 (S D 14.8). t equals 1.75, d f equals 87.

- Stigma factor Social distance: Total Mean 0.44 (S D 0.23). Mental illness group Mean 0.39 (S D 0.21). Illness group Mean 0.47 (S D 0.23). t equals 1.68, d f equals 93.

Note: Asterisks indicate significance levels where three asterisks represent p less than 0.001.

Navigate back to Table 2..

### Table 3. Long description

The table is organized into three columns: Categorical variables, Mental illness n = 52 (n and percentage), and Social distance factor (r value).

Categorical Variables:

* Gender: 14 females (26.9%) and 38 males (73.1%). Correlation r = 0.56.

* Marital status: 7 separated (13.2%), 2 single (3.8%), 39 married or cohabiting (73.6%), and 4 widowed (7.6%). Correlation r = minus 0.03.

* Education: 6 never attended (11.5%), 35 incomplete primary (67.3%), 7 complete primary (13.5%), and 4 O-Level (7.70%). Correlation r = 0.02.

* Religion: 43 Christian (82.7%) and 9 Muslim (17.3%). Correlation r = minus 0.021.

* Diagnoses: Depression 23 (44.2%), Anxiety 10 (19.2%), Schizophrenia 3 (5.8%), Substance use 15 (28.8%), Psychosis 2 (3.8%), Other 1 (1.9%), and Unknown 1 (1.9%).

* M I N I diagnoses: Alcohol use 29 (53.8%), Depression 25 (48.1%), Anxiety 19 (36.5%), Psychosis 10 (19%), Mania 9 (17.5%), Schizophrenia 2 (4.0%), Schizoaffective 1 (2.0%), and Mood 9 (17.5%).

Continuous Variables (Mean and Standard Deviation):

* Age: 45.7 (16.2) with r = minus 0.22.

* Psychometric screening tools:

- A U D I T Score: 14.5 (12.5) with r = 0.02.

- P H Q-9 Score: 13.0 (6.44) with r = 0.07.

- M D Q Score: 0.34 (0.48) with r = minus 0.19.

- G A D-7 Score: 10.5 (5.4) with r = minus 0.07.

- P S Q Score: 0.58 (0.50) with r = minus 0.39.

Significance levels are indicated by asterisks: *p < 0.05, **p < 0.01, ***p < 0.001.

Navigate back to Table 3..
