# Supplementary material for: Correlates of mental health stigma in the Buyende district of Eastern Uganda
Source: Glob Ment Health (Camb). 2026 Jul 2;13:e144. doi: 10.1017/gmh.2026.10269 (PMC13373279; doi:10.1017/gmh.2026.10269)
Supplement: Chang et al. supplementary material 1 — Chang et al. supplementary material [file S2054425126102696sup001.pdf]

Appendix Table A1

Factor Loadings from Exploratory Factor Analysis of Stigma Items

Table A1. Factor loadings from exploratory factor analysis of stigma items (n=126 respondents without mental illness)

Item Description	RW	F1	F2	F3	Single factor
Believes most people would object to mentally ill people living in their neighborhood	No	0.68	0.08	0.18	0.57
Indicates a willingness to work with someone with a mental illness*	Yes	0.66	0.07	0.14	0.52
Believes most people find it frightening to think of people with mental problems being their neighbors	No	0.55	-0.00	0.35	0.55
Believes people with mental illness are a burden on society	No	0.53	-0.10	0.45	0.53
Believes people with mental illness are a public nuisance	No	0.53	-0.12	0.44	0.51
Believes most people are afraid of people with mental illness	No	0.47	0.01	0.39	0.52
Believes most people would avoid conversations with mentally ill neighbors	No	0.46	0.28	-0.05	0.40
Afraid of people with mental illness	No	0.44	0.09	0.16	0.42
Would object to mentally ill people living in their neighborhood	No	0.42	0.08	0.06	0.34
Indicates a belief that most people would be willing to work with someone with mental illness*	Yes	0.40	0.09	0.15	0.39
Would not want to live next door to someone with a mental illness	No	0.38	0.17	-0.16	0.24
Would avoid conversations with neighbors who suffered from mental illness	No	0.37	0.15	-0.36	0.10
Indicates a belief that most people would have casual conversations with mentally ill neighbors*	Yes	0.13	0.75	0.09	0.50
Indicates a belief that most people would invite mentally ill individuals into their home*	Yes	0.11	0.72	0.19	0.53
Indicates willingness to have casual conversations with neighbors who suffer from mental illness*	Yes	0.07	0.65	0.16	0.46
Indicates a willingness to invite someone with mental illness into their home*	Yes	0.07	0.61	0.12	0.42
Indicates a belief that residents do not have something to fear from people entering neighborhoods*	Yes	0.04	0.57	-0.02	0.31
Believes most people have no sympathy for those with mental illness	No	0.04	0.37	-0.02	0.21
Believes most people would not want to live next door to someone who has been mentally ill	No	0.33	0.34	0.09	0.44
No sympathy for people with mental illness	No	0.15	0.22	0.00	0.22
Indicates willingness to include mentally ill people in the neighborhood*	Yes	-0.03	0.16	-0.14	-0.01
Believes most people believe that most women who were once patients in a mental hospital can be trusted to watch their child*	Yes	0.06	0.56	0.59	0.66
Indicates a belief that more emphasis should not be placed on protecting the public from mentally ill people*	Yes	0.04	0.18	0.58	0.45
Indicates a belief that people with mental health problems should have equal job rights*	Yes	0.27	0.15	0.58	0.58
Indicates a belief that most women who were patients in a mental hospital can be trusted to watch children*	Yes	0.10	0.48	0.49	0.59
Finds it frightening to think of mentally ill people being neighbors	No	0.41	-0.00	0.48	0.53

Item Description	RW	F1	F2	F3	Single factor
Believes anyone with mental illness should not be given responsibility	No	0.17	-0.00	0.46	0.37
Believes it is foolish for a woman to marry a man who has recovered from mental illness	No	0.06	0.38	0.39	0.46
Would exclude people with mental illness from public office	No	0.09	0.13	0.33	0.32

Note. RW = reverse-worded; F1 = Factor 1 (Fear and social distancing); F2 = Factor 2 (Avoiding personal involvement); F3 = Factor 3 (Residential proximity and trust); Single factor = loading from single-factor solution. Factor analysis used minimum residual extraction with varimax rotation. Asterisk (*) denotes items originally worded positively (indicating acceptance/willingness) that were reverse-scored so that higher values indicate greater stigma.

Among items with loadings greater than or equal to 0.40: Factor 1 contained 11 items (18% reverse-worded), Factor 2 contained 7 items (100% reverse-worded), and Factor 3 contained 8 items (50% reverse-worded). Velicer's MAP test suggested 0 factors when reverse-worded items (n=12; Cronbach's alpha=0.78) and non-reverse-worded items (n=17; Cronbach's alpha=0.78) were analyzed separately, supporting essential unidimensionality with method effects attributable to item wording direction. Overall scale Cronbach's alpha=0.86.
