# Supplementary material for: Correlates of mental health stigma in the Buyende district of Eastern Uganda
Source: Glob Ment Health (Camb). 2026 Jul 2;13:e144. doi: 10.1017/gmh.2026.10269 (PMC13373279; doi:10.1017/gmh.2026.10269)
Supplement: Chang et al. supplementary material 2 — Chang et al. supplementary material [file S2054425126102696sup002.docx]

Appendix Table A2

Factor Loadings from Exploratory Factor Analysis of Stigma Items

# **Table A2.** Factor loadings from exploratory factor analysis of stigma items (n=52 respondents with indication of mental illness

| Item Description | RW | F1 | F2 | F3 | Single factor |
| --- | --- | --- | --- | --- | --- |
| Believes most people would object to mentally ill people living in their neighborhood | No | 0.49 | 0.16 | 0.37 | 0.54 |
| Indicates a willingness to work with someone with a mental illness* | Yes | 0.51 | 0.10 | 0.37 | 0.51 |
| Believes most people find it frightening to think of people with mental problems being their neighbors | No | 0.62 | 0.07 | 0.23 | 0.55 |
| Believes people with mental illness are a burden on society | No | 0.70 | -0.02 | 0.09 | 0.50 |
| Believes people with mental illness are a public nuisance | No | 0.69 | 0.00 | 0.07 | 0.49 |
| Believes most people are afraid of people with mental illness | No | 0.58 | 0.10 | 0.06 | 0.50 |
| Believes most people would avoid conversations with mentally ill neighbors | No | 0.19 | 0.22 | 0.48 | 0.38 |
| Afraid of people with mental illness | No | 0.42 | 0.07 | 0.27 | 0.41 |
| Would object to mentally ill people living in their neighborhood | No | 0.31 | 0.04 | 0.43 | 0.33 |
| Indicates a belief that most people would be willing to work with someone with mental illness* | Yes | 0.40 | 0.11 | 0.24 | 0.41 |
| Would not want to live next door to someone with a mental illness | No | 0.14 | 0.18 | 0.41 | 0.30 |
| Would avoid conversations with neighbors who suffered from mental illness | No | 0.01 | -0.01 | 0.58 | 0.12 |
| Indicates a belief that most people would have casual conversations with mentally ill neighbors* | Yes | 0.05 | 0.72 | 0.16 | 0.52 |
| Indicates a belief that most people would invite mentally ill individuals into their home* | Yes | 0.09 | 0.73 | 0.09 | 0.54 |
| Indicates willingness to have casual conversations with neighbors who suffer from mental illness* | Yes | 0.10 | 0.64 | 0.04 | 0.49 |
| Indicates a willingness to invite someone with mental illness into their home* | Yes | 0.04 | 0.63 | 0.09 | 0.45 |
| Indicates a belief that residents do not have something to fear from people entering neighborhoods* | Yes | -0.05 | 0.51 | 0.08 | 0.31 |
| Believes most people have no sympathy for those with mental illness | No | 0.03 | 0.28 | 0.03 | 0.21 |
| Believes most people would not want to live next door to someone who has been mentally ill | No | 0.17 | 0.42 | 0.19 | 0.44 |
| No sympathy for people with mental illness | No | 0.05 | 0.17 | 0.09 | 0.17 |
| Indicates willingness to include mentally ill people in the neighborhood | Yes | -0.06 | 0.09 | 0.07 | 0.03 |
| Believes most people believe that most women who were once patients in a mental hospital can be trusted to watch their child* | Yes | 0.33 | 0.70 | -0.24 | 0.63 |
| Indicates a belief that more emphasis should not be placed on protecting the public from mentally ill people* | Yes | 0.40 | 0.32 | -0.22 | 0.45 |
| Indicates a belief that people with mental health problems should have equal job rights* | Yes | 0.60 | 0.27 | -0.06 | 0.60 |
| Indicates a belief that most women who were patients in a mental hospital can be trusted to watch children* | Yes | 0.32 | 0.60 | -0.15 | 0.59 |
| Finds it frightening to think of mentally ill people being neighbors | No | 0.64 | 0.08 | 0.05 | 0.53 |

| Item Description | RW | F1 | F2 | F3 | Single factor |
| --- | --- | --- | --- | --- | --- |
| Believes anyone with mental illness should not be given responsibility | No | 0.46 | 0.15 | -0.09 | 0.42 |
| Believes it is foolish for a woman to marry a man who has recovered from mental illness | No | 0.20 | 0.52 | -0.12 | 0.46 |
| Would exclude people with mental illness from public oﬀice | No | 0.25 | 0.28 | -0.06 | 0.35 |

*Note.* RW = reverse-worded; F1 = Factor 1 (Fear and social distancing); F2 = Factor 2 (Avoiding personal involvement); F3 = Factor 3 (Residential proximity and trust); Single factor = loading from single-factor solution. Factor analysis used minimum residual extraction with varimax rotation. Asterisk (*) denotes items originally worded positively (indicating acceptance/willingness) that were reverse-scored so that higher values indicate greater stigma.

Among items with loadings greater than or equal to 0.40: Factor 1 contained 11 items (18% reverse-worded), Factor 2 contained 7 items (100% reverse-worded), and Factor 3 contained 8 items (50% reverse-worded). Velicer’s MAP test suggested 0 factors when reverse-worded items (n=12; Cronbach’s alpha=0.78) and non-reverse-worded items (n=17; Cronbach’s alpha=0.78) were analyzed separately, supporting essential unidimen- sionality with method effects attributable to item wording direction. Overall scale Cronbach’s alpha=0.86.
