# Supplementary material for: Correlates of mental health stigma in the Buyende district of Eastern Uganda
Source: Glob Ment Health (Camb). 2026 Jul 2;13:e144. doi: 10.1017/gmh.2026.10269 (PMC13373279; doi:10.1017/gmh.2026.10269)
Supplement: Peer review history

# Reviewer report: Review: Correlates of mental health stigma in the Buyende district of Eastern Uganda — R0/PR2

**DOI:** 10.1017/gmh.2026.10269.pr2

Thank you for the opportunity to review for the journal. This manuscript describes the findings of a cross-section study of mental illness stigma amogn 178 adults in a rural area of Eastern Uganda, a context where formal mental health services are underfunded and cultural factors strongly shape care-seeking. The authors administered 33 items from a variety of stigma scales, conducted exploratory factor analysis, and then estimated correlates of mental illness stigma while also comparing people with vs. without mental illness. They found that (1) stigma items cluster into three factors: fear/social distancing, avoidance of close personal involvement, and avoidance of residential proximity/conversation; (2) in the full sample, the only robust correlate of higher stigma is that women have higher levels of stigmatizing attitudes; and (3) within the mental illness subgroup, women have higher levels of fear and relationship-focused stigma, while Muslims are more likely to report residential separation. The authors interpret these results as suggesting that gendered social roles and, to a lesser extent, religious context are key correlates of stigma, with implications for targeted anti-stigma interventions. The central concerns relate to conceptual questions about what the authors are actually measuring in the two subgroups, limitations and clarity of the sampling and case-definition strategy, and the adequacy and interpretation of the factor analysis.

Major comments:

1. I assume the authors administered the same items to the people with mental illness vs. the people without mental illness. What does it mean when a person with a mental illness diagnosis responds affirmatively to an item like “I am afriad to think of mentally ill people being neighbors” or “people with mental illness are a burden on society”? Conceptually, when a person with a stigmatized condition responds affirmatively to a negative attitude held in society, that suggests that the person with the stigmatized condition has internalized that attitude and applied it to the self-- which is exactly the definition of internalized stigma (or self stigma). This is akin to the “parallel scales” approach that Visser et al. used in measuring HIV stigma (AIDS Behav. 2008;12:759-71) and the approach that the PopART team used much later (Stangl et al. J Int AIDS Soc 2019;22:e25421). The authors here basically apply a uniform EFA to the entire sample and then assess for differences between the mentally ill vs. non-mentally ill subgroup. But that seems like putting the cart before the horse because it assumes that the constructs are the same in the two populations.

2. Sampling strategy: The sample mixes convenience-recruited community members and clinic/VHT-referred individuals, which is appropriate for an exploratory study but substantially complicates any comparisons about “the general population” vs. “the mental illness subgroup.” The mentally ill group is defined by positive screens plus MINI modules, but the text is not fully transparent about whether all screen-positive participants met MINI criteria or how discordant cases were handled. My guess is that if someone screened positive on the PSQ (say) but did not meet criteria on any of the MINI modules, then they would not have been included in the study; but that is just a guess. The strong sex differences between groups (26.9% women in the mental illness group vs 63.5% in the non-mental illness group) raises concern about selection bias: for example, differential help-seeking/access or recruitment channels by sex. Because sex also comes out of the regression models as the main explanatory variable for stigma, these selection processes may severely bias estimates and the interpretation that “female gender is the strongest correlate of stigma.” The paper needs a much more explicit description of: (a) recruitment flows for each subgroup; (b) the proportion of screen-positive participants who did and did not meet MINI criteria; and (c) how these processes could have produced the observed sex differences in both exposure (mental illness) and outcome (stigma). The Discussion should be more cautious about generalizing beyond a highly selected and small clinical subgroup.

3. Analysis: There are several issues here. First, conceptually speaking all 3 of the factors identified are essentially the same thing. Factor 1, “fear & social distancing” (I am afraid of people with mental illness, people don’t want the mentally ill in their neighborhoods). Factor 2, “avoiding personal involvement” (I don’t want them in my home, people don’t want to live near the mentally ill). Factor 3, “avoiding residential proximity” (I don’t want the mentally ill in my neighborhood, people don’t want to talk with the mentally ill). These itemes are all essentially nibbling at the edges of the same construct, which is social distancing, i.e., separation. There is really not enough detail in the frst paragraph of the Results that would help me understand the extent to which the exploratory factor analysis was well conducted. I don’t see key diagnostics (e.g. KMO, Bartlett’s test, item response scales), factor inter-correlations, etc. Based on the items themselves, I would have expected just a single dominant factor to emerge from the EFA, or possibly one primary factor and then some modest evidence for a third factor. But on its face, the 3 factor solution doesn’t really make sense to me. The authors mention “orthogonal factor analysis”, which I assume means they used orthogonal rotation. But an oblique rotation would be more appropriate (because the stigma dimensions are likely correlated, if in fact there are multiple dimensions).

Additionally:

(a) Conducting an exploratory factor analysis of 33 items with N=178 is at the lower limit of acceptability, especially given that all of the items are dichotomous. An EFA based on Pearson correlations and standard methods is not ideal-- better would be a tetrachoric or polychoric correlation matrix with an estimator suitable for categorical data would be preferred.

(b) With dichotomous items, the information per item is reduced relative to (for example) ordinal Likert-type items. That pushes the design even closer to the lower bound of an acceptable N for a 33‑item EFA. This does not necessarily invalidate the results, but it does mean the factor structure should be interpreted very cautiously. The low Cronbach’s alpha (0.59) obtained for Factor 3 reinforces the concern with dichotomous items, because alpha is already somewhat constrained by item format. Conclusions based on Factor 3 (e.g., the muslim/residential separation finding) should be interpreted more cautiously (again, subject to my concern about whether there are even 3 factors to begin with).

(c) With the caveat that my comments about the stratified analysis may fall apart depending on how the revised EFA looks: fitting a multivariable regression model (I didn’t count the number of variables) to the data is ambitious given the mental illness subgroup sample size (N=52) and the multiple stigma outcomes.

Points of detail:

4. The authors should provide the full set of regression coefficients in tables. That would help me understand exactly what was done. I think (but am not sure) that they fit one set of multivariable regression models for the “non-mental illness group” (N=126) and one set of multivariable regression models for the “mental illness subgroup” (N=52). Is that correct? If so, that should be directly stated somewhere.

5. In general, the exact P-values should be reported with a floor of “P<.001”. There is no reason to write “P<.03”, for example.

6. The interaction analysis reporting (e.g., t=1.89 with P<.03) seems inconsistent with conventional two-sided P-values. Under a conventional two-sided t‑test, with t=1.89 and df=79 (I could be off), the two‑sided P‑value would be roughly P=.06 (a bit under .07, but clearly >.05). Did the authors actually use a one‑sided P‑value for that same t? That would put it closer to P=.03. Please recheck here and throughout.

7. The authors state that individuals with missing or incomplete data were excluded “from individual analyses depending on availability of data,” but no details are given on the extent or pattern of missingness. A simple appendix table describing item- and variable-level missingness and the number included in each analysis would help readers assess potential bias.

8. I assume that each of the items were dichotomous. The authors point to other manuscripts but do not directly state that these are dichotomous items. That should be mentioned somewhere.

9. The paper could benefit from a brief comparison of the three factors-- again, with the caveat that I am not completely convinced there are 3 factors-- with established dimensions of stigma (e.g., public stigma, self-stigma, etc).

10. There are some minor typographical errors (e.g., “behaviora patterns”) and inconsistencies in reference formatting (e.g., some in-text citations lack consistent punctuation or ordering).

# Reviewer report: Review: Correlates of mental health stigma in the Buyende district of Eastern Uganda — R0/PR3

**DOI:** 10.1017/gmh.2026.10269.pr3

Thank you for the opportunity to review this manuscript, it is an interesting contribution to the literature and an area worthy of research. However, the manuscript on the whole is difficult to follow and there are several gaps within the manuscript which make both the results and the discussion difficult to understand. There is limited information around cut-offs, validity of items, rationale for why decisions were made and transparency for replicability. Please read full manuscript and check for typos.

Impact statement

Remove particularly from this sentence: “Particularly, rural settings within LMICs are 27 especially vulnerable due to a lack of formal mental health services.”

This sentence does not make sense: “…while identifying 29 noteworthy high-risk subgroups that may need specific intervention risk factors that may need special attention.”

Introduction

When you say there are various studies examining mental illness stigma in Uganda, can you provide more information on what they found and also references?

The final paragraph of your introduction includes information around the methodology that could be omitted here and included within the methods section instead, ensuring the final paragraph of the introduction clearly sets out the aims of the study. It may be clearer to have the aims broken up and numbered. I currently feel as though the final paragraph is cluttered and unclear.

Provide a definition of stigma.

Methods

Please provide full details of ETH abbreviation.

Information on sample e.g. number of participants and demographics within text and participant section is lacking.

Provide a bit more information on M.I.N.I assessments.

It would be good to see information regarding the validity and reliability of the measures used in the study, for example reporting Cronbachs alpha.

More information on the measures in general would be helpful, such as example items.

Why did you decide to use three different sources to measure stigma? What informed this decision and why did you pick the items that you did etc?

The information around the measures is extremely limited, please ensure there is transparency around why decisions were made to use a range of measures and the validity of these.

Provide rationale around why you have decided to do a factor analysis.

How did you decide on the cut-offs?

There is literature stating that fixed rules of thumb e.g. >0.40 are sample dependent and should not be treated as absolute, statistical and contextual judgement should be used alongside these to decide upon item retention. There is a lack of discussion around how this cut-off was decided upon and if contextual/statistical judgement was used.

# Reviewer report: Review: Correlates of mental health stigma in the Buyende district of Eastern Uganda — R0/PR4

**DOI:** 10.1017/gmh.2026.10269.pr4

Overall, this is a strong paper on an interesting topic. I would suggest to especially clarify the methods section.

Introduction

- It would be helpful to clarify what mental illnesses authors are referring to. For example, is the study seeking to evaluate associations with sociodemographic characteristics long with a range of symptoms? If so, it would be helpful in the first few paragraphs of the introduction section to also cite existing evidence on if and how stigma is different for invisible or visible mental illness symptoms.

- In the last paragraph, rather than specifying the analysis, which should be mentioned in the Methods section, I would suggest the authors to add the objectives of the study and research questions that they are hoping answer.

Methods

- Study site: It is unclear what authors mean by “complex patterns of care-seeking”. It would be helpful to state existing evidence related to care seeking, stigma and mental health in this region.

- Study design: How were participants of the survey chosen by the VHTs? How were individuals recruited from the patient cohort, especially those with mental illnesses? Which types of mental illnesses?

- Study Design and Participants: Did authors aim to recruit a certain number (or percentage) of people who identified as having a mental illness?

- Informed consent and participant protections: If participants were identified as needing mental health support, were they referred to any existing services?

- Measures, Stigma Items: It would be helpful to explain what the different groups of items in the stigma scale measure.

- Measures: What do the different modules of the MINI address and measure?

- Measures: What sociodemographic characteristics were included? What range of mental illness symptoms were included to establish correlates?

- Analysis: Were they participants that met the screening criteria for a mental disorder through the various screeners but was not confirmed by the MINI? Were they included with those that did not meet criteria for a mental disorder for the bivariate analysis?

Results and Discussion

- What were the demographics of the 178 respondents? (by gender, age, mental illness)

- Clear description of results

# Editor report: Recommendation: Correlates of mental health stigma in the Buyende district of Eastern Uganda — R0/PR5

**DOI:** 10.1017/gmh.2026.10269.pr5

**Affiliations:** The George Institute for Global Health India, India

Please respond to the reviewers' comments and resubmit.

# Editor report: Decision: Correlates of mental health stigma in the Buyende district of Eastern Uganda — R0/PR6

**DOI:** 10.1017/gmh.2026.10269.pr6

**Affiliations:** London School of Hygiene & Tropical Medicine, United Kingdom of Great Britain and Northern Ireland

No accompanying comment.

# Reviewer report: Review: Correlates of mental health stigma in the Buyende district of Eastern Uganda — R1/PR8

**DOI:** 10.1017/gmh.2026.10269.pr8

GMH-2026-0001.R1

Correlates of Mental Health Stigma in the Buyende District of Eastern Uganda

Thank you for the opportunity to review this revised manuscript. In general, the authors were responsive to my major points. I would be happy to recommend acceptance at this point.

There are a few remaining points that I would regard as optional. I make these comments really only for the purposes of making improvement tweaks. I would leave it up to the editor’s discretion as to whether these should be addressed before accepting the manuscript for publication. (If you send this manuscript back to the authors for a second round of revisions, and then send it back to me, then I will more or less automatically accept whatever revisions they make.)

1. In my original comment #1, I raised the concern that identical items administered to people with vs without mental illness do not necessarily measure the same construct. For people with mental illness, agreement with statements like “people with mental illness are a burden on society” is usually interpreted as internalized/self stigma, whereas for people without mental illness the same items capture personal or perceived public stigma. In the new Limitations paragraph, tghe authors now state that “our findings of social distancing stigma reflect internalized stigma rather than attitudes towards public stigma... responses captured self-directed beliefs about mental illness rather than societal attitudes.” As written, this is not really accurate for the full sample: among the 126 respondents without evidence of mental illness, these items do *not* reflect internalized stigma. Rather, they capture negative attitudes toward people with mental illness in general (or what Corrigan might call “public stigma”).

The appendices show that the factor structure is essentially the same in the non-mental-illness and mental-illness subgroups, which is reassuring regarding measurement consistency. But sameness of factor loadings does not resolve the conceptual distinction in what the score *means* in those two groups.

At a minimum, I would suggest that in the Limitations and Discussion the authors specify that, for respondents without evidence of mental illness, the factor has to do with negative attitudes toward people with mental illness (public stigma); and for respondents with mental illness, it more closely approximates internalized stigma. Then they would accordingly soften the current statement that “our findings of social distancing stigma reflect internalized stigma rather than attitudes towards public stigma” to something like: “Among respondents with evidence of mental illness, endorsement of these items likely reflects internalized stigma; among respondents without mental illness, the same items reflect public stigma. Our pooled analyses therefore combine related but conceptually distinct stigma processes, which we cannot separate given the current design.”

2. My earlier suggestion was to use a tetrachoric (or polychoric) correlation matrix with an estimator suited to categorical data. The revised methods refer to “minimum residual extraction with varimax rotation” but do not say whether correlations were Pearson or tetrachoric. My reading is that standard Pearson correlations were used. With 29 dichotomous items and N=178, this is defensible but not ideal; factor loadings and explained variance will be somewhat attenuated, and the sample-size concerns I previously raised still apply. At a minimum, I think the authors should (a) explicitly state the type of correlation matrix used, and (b) add one or two sentences acknowledging that using standard EFA methods for dichotomous items is a limitation and that the unidimensional structure should be interpreted cautiously.

3. The note under Table 1 still refers to “orthogonal analysis” and varimax rotation even for the single-factor solution. For a unidimensional model, I don’t thiknk rotation is relevant, is it? This is only a wording issue, but it might confuse some readers. The authors could simply state that the final single-factor solution was estimated without rotation.

4. The revised methods provide a clearer description of recruitment flows, including the role of VHTs and the Empower Through Health clinic, the use of the screening instruments plus MINI, and the handling of screen-positive / MINI-negative participants. However, the very large gender imbalance between the mental-illness (73.1% male) and non-mental-illness groups (63.5% female) remains only briefly noted in the text. Because gender is also the main correlate of stigma in all analyses, I still worry about selection mechanisms. Clinic- and VHT-based recruitment may over-represent men with more severe or disruptive presentations, or differential help-seeking patterns by gender. Community convenience sampling may have preferentially recruited women (e.g., if interviews were conducted when men were more likely to be out of the home). The m anuscriopt would therefore be more complete if it included a somewhat more explicit discussion of possible selection processes and how they might bias the gender comparisons. For example, a short paragraph in the Limitations could note that the mental-illness subgroup is a small, highly selected clinical sample with different gender composition than the community comparison group. It could also mention that if women with mental illness are under-represented among clinic attenders (relative to community prevalence) but over-represented among community respondents, the observed association between female gender and higher stigma might partially reflect these recruitment patterns rather than true population differences.

5. The shift to a single stigma factor resolves my earlier concern about fitting multiple multivariable models in a small N=52 subgroup. However, even with one outcome, the stepwise multivariable regression in the mental-illness subgroup includes a fairly large set of candidate predictors (sociodemographics, self-reported diagnoses, MINI diagnoses, and continuous symptom scores). With N=52, any model with more than a handful of predictors risks overfitting and unstable coefficients. I would suggest the authors emphasize that the multivariable findings in the mental-illness subgroup are exploratory and should be interpreted cautiously due to the small sample.

Also, I generally think it is good to have a table with the full set of regression coefficients, confidence intervals, and p-values (at least for the mental-illness subgroup), e.g. in an appendix. At present, some coefficients are only given in text, and nonsignificant terms are not reported.

6. In the factor analysis section, the sentence "Velicer’s MAP suggested 0 factors for both reverse-worded items... and non-reverse-worded items..., consistent with unidimensionality within wording type“ might puzzle some readers, since ”0 factors“ sounds like ”no latent structure at all." I think the authors mean no additional factors _beyond_ a general factor (which is consistent with unidimensionality).

7. There are a few places where the authors write "p = <0.001". I don’t know what this means. I think they mean “p < 0.001”.

8. FYI in the reference list, the Sheehan et al. citation runs together with the Spitzer et al. GAD-7 citation in one block of text.

# Editor report: Recommendation: Correlates of mental health stigma in the Buyende district of Eastern Uganda — R1/PR9

**DOI:** 10.1017/gmh.2026.10269.pr9

**Affiliations:** The George Institute for Global Health India, India

Please see some additional comments from the reviewer to strengthen the paper. Please revise accordingly and resubmit

# Editor report: Decision: Correlates of mental health stigma in the Buyende district of Eastern Uganda — R1/PR10

**DOI:** 10.1017/gmh.2026.10269.pr10

**Affiliations:** London School of Hygiene & Tropical Medicine, United Kingdom of Great Britain and Northern Ireland

No accompanying comment.

# Editor report: Recommendation: Correlates of mental health stigma in the Buyende district of Eastern Uganda — R2/PR12

**DOI:** 10.1017/gmh.2026.10269.pr12

**Affiliations:** The George Institute for Global Health India, India

No accompanying comment.

# Editor report: Decision: Correlates of mental health stigma in the Buyende district of Eastern Uganda — R2/PR13

**DOI:** 10.1017/gmh.2026.10269.pr13

**Affiliations:** London School of Hygiene & Tropical Medicine, United Kingdom of Great Britain and Northern Ireland

No accompanying comment.
